# Influence and Role of Regulatory B Cells in Organ Transplantation: The State of the Art, Prospects, and Emerging Insights

**DOI:** 10.3390/antib14040095

**Published:** 2025-11-07

**Authors:** Marina Fernández-González, Santiago Llorente, José Antonio Galián, Carmen Botella, Rosana González-López, María José Alegría, Alicia Hita, María Rosa Moya-Quiles, Helios Martinez-Banaclocha, Manuel Muro-Pérez, Javier Muro, Alfredo Minguela, Isabel Legaz, Manuel Muro

**Affiliations:** 1 Immunology Service, University Clinical Hospital Virgen de la Arrixaca, Biomedical Research Institute of Murcia (IMIB), 30120 Murcia, Spain; marina.fernandez3@carm.es (M.F.-G.); josea.galian3@carm.es (J.A.G.); mcarmen.botella@carm.es (C.B.); rosana.gonzalez@carm.es (R.G.-L.); mariaj.alegria@carm.es (M.J.A.); alicia.hita@carm.es (A.H.); rosa.moya2@carm.es (M.R.M.-Q.); helios.martinez2@carm.es (H.M.-B.); manuel.murop@um.es (M.M.-P.); javimuro12@gmail.com (J.M.); alfredo.minguela@carm.es (A.M.); 2 Nephrology Service, University Clinical Hospital Virgen de la Arrixaca, Biomedical Research Institute of Murcia (IMIB), 30120 Murcia, Spain; santiagoj.llorente@carm.es; 3 Department of Legal and Forensic Medicine, Biomedical Research Institute of Murcia (IMIB), Faculty of Medicine, Regional Campus of International Excellence “Campus Mare Nostrum”, University of Murcia, 30100 Murcia, Spain; isalegaz@um.es

**Keywords:** kidney transplant, regulation of immune response, regulatory B cells, tolerance, transitional B cells

## Abstract

B cells have attracted increasing interest in the field of organ transplantation due to their newly discovered immunoregulatory properties in alloimmune responses. Traditionally, B cells have been primarily associated with adaptive immunity to foreign substances and alloreactive immune response to allografts, differentiating into antibody-producing plasma cells or memory cells upon antigen recognition and T cell collaboration. However, the existence of B cells with regulatory functions (Bregs) in humans has been widely confirmed, highlighting the presence of this subset, which has immunosuppressive properties and which might contribute to allograft tolerance, within the B cell compartment in humans and mice. In this mini review, we summarize all the information available in the published reports about the role of regulatory B cells in solid organ transplantation.

## 1. Introduction

Rejection is one of the leading causes of dysfunction and loss of graft in solid organ transplantation. According to the time of onset, three main types of rejection can be distinguished: hyperacute rejection, which takes place immediately after transplantation and is caused by the existence of preformed donor-specific antibodies (DSAs) in the recipients; acute rejection, which usually occurs after the first week post-transplantation and can be mediated by T cells, macrophages, and/or DSAs; and chronic rejection, typically emerging within months or years after organ transplantation [1,2]. Although the precise mechanisms underlying chronic rejection are not fully understood, since Dr. Terasaki released his humoral theory of transplantation in 2003, it has been widely recognized that the development of DSAs plays an important role [3].

A variety of therapeutic approaches are currently employed to manage humoral rejection to reduce the production of DSAs and minimize damage to the allograft, including plasma exchange (plasmapheresis or immunoadsorption), intravenous immunoglobulin administration, and anti-CD20 or anti-CD38 monoclonal antibody and anti-proteasome inhibitor immunotherapies. These treatments have shown acceptable results in acute humoral rejection, yet their efficacy to treat chronic humoral rejection remains limited, compromising long-term graft survival [4]. Data from the National Scientific Registry of Transplant Recipients in the US show that while overall one-year post-kidney transplant survival rates are excellent (approximately 95%), ten-year survival rates barely reach 70% [5]. In this context, there arises a strong and urgent need to find novel biomarkers capable of risk-stratifying patients and predicting rejection before graft failure.

In recent years, the role and influence of B cells have gained increasing interest in different fields of pathologies and organ transplantation due to their newly discovered immunoregulatory properties. They are reported to modulate inflammatory responses independently of antibody production [6]. B cells have been principally associated with immune responses against infectious pathogens (viruses, bacteria, fungi, and parasites), allergic and autoimmune processes, and alloreactive responses to allografts, differentiating from plasmablast to antibody-producing plasma cells (PCs) or memory B cells upon foreign antigen identification and presentation with the necessary help of the T cell compartment [6]. However, in 2009, Clatworthy et al. published the first evidence supporting the existence of B cells with regulatory functions in human transplantation [7]. They observed that renal transplant recipients treated with an anti-CD20 monoclonal antibody as induction therapy had higher rates of acute rejection than both control patients and those with no induction therapy at all [7]. As this fact is widely used in transplantation, induction therapy with an anti-CD20 is generally used in organ and stem cell transplantation to deplete B cells and reduce the risk of rejection or complications like post-transplant lymphoproliferative disorder. However, its effectiveness and routine use vary by transplant type and patient risk factors [7]. Ten years later, a study in heart transplant recipients similarly reported that patients receiving rituximab at the time of transplant exhibited increased cardiac allograft vasculopathy one year after transplantation [8]. These results underlined the presence of a subset with immunosuppressive properties within the B cell compartment in humans that might contribute to allograft tolerance [9].

In general, B cell phenotypes refer to the distinct cell surface marker patterns that define different types of B cells, such as naïve B cells (CD19^+^, CD20^+^, IgD^+^), memory B cells (CD19^+^, CD20^+^, IgD^−^, CD27^+^), and plasma cells/blasts (CD19low, CD20−, CD138+), which can be identified using flow cytometry [9]. These phenotypes are crucial for understanding B cell development and function in the immune response. They are also used to diagnose and monitor diseases like immunodeficiency and autoimmune conditions [10,11]. B cell development follows a series of stages, each with a unique set of markers: (1) *immature B cells* develop from hematopoietic stem cells through progenitor B and precursor B cells; (2) *transitional B cells* are a short-lived intermediate stage of B cells that are immature but have survived selection; (3) *naïve B cells* are cells that have not encountered their specific antigen and express CD19, CD20, and high levels of IgD; (4) *memory B cells,* upon antigen encounter, can differentiate into memory B cells, which express CD19, CD20, and CD27, but are typically IgD^−^; and (5) *plasma cells* are terminally differentiated B cells that have lost most surface markers (like CD20) but gain markers like CD38 (Syndecan-1), CD138, and CD24 and are responsible for antibody secretion [12,13].

Regarding the arrangement of Bregs in the B cell compartment, it is important to know that several groups have demonstrated the presence of a particular B cell subset producing the IL-10 cytokine (with wide immunosuppressive effects) and have been named as regulatory B cells or Bregs [9,10]. In non-pathological situations, these Breg cells are at scarce or very discrete levels, and their function is to maintain states of immune tolerance and the correct homeostasis of the system [10,11]. They are preferably activated in inflammatory phenomena and exacerbations of the immune response, whether autoimmune, oncological, or of another nature, where a hyper-response occurs and where Bregs must be present to manifest their potential for necessary immunosuppressive functions [11,12]. Bregs will be differentiated and activated by multiple signals and interactions with specific molecules essential in immune responses, such as Toll-like receptor (TLR)-2, TLR-4, TLR-9 signaling, B cell receptor (BCR) signaling, and costimulation mediated by CD40, CD80/CD86, or B cell activating factor (BAFF) and different cytokines [IL-1β (IL, interleukin), IL-2, IL-6, IL-10, IL-17, IL-21, IL-27, IL-35, IFN-α/β/γ (IFN, interferon) or TNF-α (Tumor Necrosis Factor), and TGF-β (Transforming Growth Factor), among others] [9,10,11,12,13].

Although Bregs were first reported and analyzed in animal models in the field of autoimmunity and infection and later in the field of cancer (and a similar fate, in terms of several aspects, exists for regulatory B cells in both mice and humans), their proper role in transplantation is still uncertain and undetermined [9,13,14]. In addition, it has also been corroborated that immature B cells, mature B cells, and plasmablasts all have the eventual capacity to differentiate into Breg cells in both animal models and humans [13].

In this mini review, we summarize all the information in the published reports about the role of regulatory B cells (Bregs) in solid organ transplantation.

## 2. Phenotypes of Regulatory B Cells

Multiple research groups have worked on the suitable characterization of Breg cells in the last decade. Nevertheless, no consensus definition or standardized classification has been reached despite substantial efforts. Instead, Bregs are considered a diverse population with immunoregulatory properties, mainly identified by their ability to secrete immunosuppressive cytokines such as IL-10, IL-35, and TGF-β [11].

Regarding their origin, the most accepted hypothesis is that they arise from B cells at different stages of development under certain conditions, as no specific lineage marker or transcription marker has been discovered, unlike Foxp3 and regulatory T cells (Tregs). In this sense, cytokines, inflammation, and cell signaling through CD40 and BCR are some stimuli that can trigger their expansion [9].

The transitional immature B cell compartment is thought to be vastly enriched in Bregs (CD19^+^CD24^hi^CD38^hi^), but they are also present within the mature B cell and plasmablast compartments (CD24^hi^CD27^+^ and CD24^hi^CD27^int^, respectively) [11,12,13]. Thus, all B-lineage cells (immature, mature, or plasmablasts) with different phenotypes and characteristics can differentiate into Breg cells [13]. Perhaps the primary requisite for Breg cell differentiation is not the expression of a Breg-cell-specific lineage marker but rather the environment in which a B cell finds itself and its eventual interactions with other cellular types and/or soluble factors [13].

**Figure 1:**
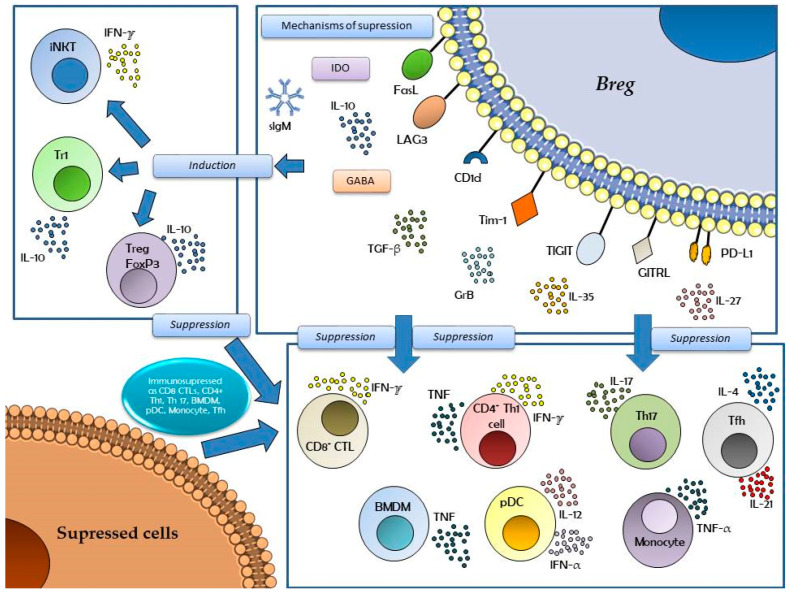
Different mechanisms exerted by Breg cells induce and suppress different immune responses. These cells respond to a variety of signals that share their pro-inflammatory profile, such as stimulation of the BCR, TLRs (such as TLR4, TLR7, or TLR9), ligands such as CD40L, costimulatory signals such as the CD80/CD86 couple, or other signaling molecules such as APRIL. Induction and suppression phenomena are mediated by cytokines such as IFN-α, IFN-γ, IL-6, IL-10, IL-21, IL-27, IL-35, TNF, TGF-β, GrB, or IL-17, and apoptotic cells (IDO) (upper right square). The signals that induce Bregs can also be influenced by the distribution of the intestinal microbiota and its metabolites (e.g., butyrate), which will determine the release of neurotransmitters (GABA) involved in the transcription of the immunosuppressive cytokine IL-10. Breg cells also induce other cells as iNKT, Treg (Foxp3^+^), and Tr1 cells (upper left square), which in turn also suppress many other cells (CD8^+^ CTL, BMDM, Th1, Th17, Tfh, pDC, or monocytes) (lower right square), via IL-10 and IFN-γ cytokines, and Breg cells directly suppress the activation of the cellular immune response through IL-10, IL-35, TGF-β, GrB, IL-27, and different molecules with a known immunomodulatory profile, such as FasL, PD-L1, CD1d, Lag-3, Tim-1, TIGIT, or GITRL, among others (upper right square). Abbreviations: DC, dendritic cell; BMDM, bone marrow-derived macrophage; Th, T helper cell; Tfh, T follicular helper; TLR, Toll-like receptor; Breg, regulatory B cell; BCR, B cell receptor; CTL, cytotoxic T lymphocyte; iNKT, Invariant Natural Killer T cell; Treg, regulatory T cell; Tr1, T regulatory Type 1 (Tr1); GrB, granzyme B; IFN, interferon; TNF, Tumor Necrosis Factor; TGF, Transforming Growth Factor; IDO, Indoleamine 2,3-Dioxygenase; GABA, gamma-aminobutyric acid; CD, cluster of differentiation; Ig, immunoglobulin; FasL, Fas ligand; LAG3, lymphocyte-activation gene 3; Tim-1, T-cell immunoglobulin and mucin domain 1; TIGIT, T cell immunoreceptor with Ig and ITIM domains; GITRL, GIT Receptor Ligand (G protein-coupled receptor kinase-interacting protein receptor ligand); PD-L1, Programmed Death-Ligand 1.

For a better understanding of the different pathways, mechanisms, and signals in Bregs, we can look at Figure 1. This graphic shows the different mechanisms that Breg cells exert to induce and suppress different immune responses. Bregs respond to a variety of signals that share their pro-inflammatory profile and immune stimulation, such as those mentioned above (BCR, TLRs, or B7 costimulatory complex), ligands such as CD40L, or other signaling molecules such as APRIL (a proliferation-inducing ligand). Induction and suppression phenomena are mediated by cytokines, such as those mentioned previously, and by apoptotic cells [mediated by IDO (Indoleamine 2,3-Dioxygenase), involved in tryptophan metabolism]. The signals that induce Bregs can also be influenced by the distribution of the intestinal microbiota and its metabolites (e.g., butyrate), which will determine the release of neurotransmitters (GABA, Gamma-aminobutyric acid) involved in the transcription of the immunosuppressive cytokine IL-10.

Breg cells also induce iNKT (Invariant Natural Killer T) cells, Treg (Foxp3^+^) and Tr1 (Type 1 regulatory) cells, which in turn also suppress many other cells (via IL-10 and IFN-γ). Breg cells directly suppress the activation of the cellular immune response through IL-10, IL-35, TGF-β, GrB (Granzyme B), IL-27, and different molecules with a known immunomodulatory profile, such as FasL (Fas-Ligand), PD-L1 (Programmed Death-Ligand 1), CD1d, Lag-3 (lymphocyte-activation gene 3), Tim-1 (T-cell immunoglobulin and mucin domain 1), TIGIT (T cell immunoreceptor with Ig and ITIM (Immunoreceptor tyrosine-based inhibition motif domains), or GITRL [G protein-coupled receptor kinase-interacting protein (GIT) receptor ligand], among others [11,14,15].

Therefore, it is crucial to note that the proper role of Breg cells in the immunosuppression of inflammatory responses may have been influenced and perhaps confused by the fact that there are multiple Breg cell subsets. Accordingly, different regulatory B cell subtypes and their possible functions in humans are shown in Table 1. Other, additional Breg cells in mice are not contemplated in this present review, including the subsets T2-MZP, MZ, FO, B10, B1a, CD1d^hi^, GABA^+^ subset, GITRL, or TIGIT [11,13].

## 3. Regulatory B Cells in Several Pathologies

Firstly, the potential implication of B cells in general and Bregs in different processes and pathologies is shown in Table 2.

Accordingly, in normal situations and in a homeostasis state, IL-10 expression by normal B cells is relatively low (3–4%) compared to its expression in Bregs, although in B cells in an immature state, it can be increased by stimulation of several signals, such as CD40 or TLR9 [20]; in consequence, it may acquire a modulatory and regulatory function, such as suppressing the differentiation of several cellular types, such as CD4^+^IFNγ^+^ and CD4^+^TNF^+^ T cells, and promoting the transition of these cells to the Treg cell phenotype [20,21]. In scientific communications, immature Breg cells with IL-10 expression are decreased in some autoimmune diseases, such as systemic lupus erythematosus (SLE), rheumatoid arthritis, or multiple sclerosis, among others, as reported in several studies; in conditions where immune system dysregulation and disruption of the correct state of homeostasis are actually key; and in those where potential specific environmental signals may also further determine the polarization, percentage, and function of B-lineage regulatory cells [20,21,22].

In this context, a 2011 study by Iwata et al. [23] showed that patients suffering from multiple sclerosis disease with a relapsing pathology showed decreased levels of intestinal bacteria bound to IgA immunoglobulin, indicating that these immature Breg cells could be involved in avoiding neuro-inflammation and therefore mediating brain protection, as reported [24,25]. Another study also showed that these cells expressed IL-10 transcripts and co-localized with inflammatory lesions in the brains of patients with multiple sclerosis. It has also been reported that, in human peripheral blood, they produce IL-10 interleukin and suppress T cell and macrophage responses through IL-10 and programmed cell death ligand 1 (PD-L1) [26]. Thus, the differentiation of IgA^+^IL-10^+^PD-L1^+^B cells requires the APRIL molecule and IL-21 interleukin, which are necessary for B cells’ survival, proliferation, and differentiation [27,28]. In this context, systemic lupus erythematosus patients also showed low numbers of circulating Bregs and iNKT cells, and anti-CD20 therapy restored iNKT cell levels following Breg repopulation [29].

On the other hand, the inhibitory marker PD-L1 also plays a crucial role in promoting immune tolerance by binding to PD-1 expressed on several immune cell subsets [30]. Like other Breg subsets, PD-L1^hi^ Bregs are primarily contained within the T2-MZP (Marginal Zone Precursor (B Cell)), MZ (Marginal Zone (B Cell), and PC B cell subsets, whilst in humans, they are found in naïve and CD24hiCD38hi-immature B cells [19,31]. Indeed, PD-L1^+^ Bregs suppressed autoimmune-driven immune responses by reducing TFH frequencies and antigen-specific antibody production [19]. Therefore, the important role of Breg cells in autoimmune and auto-inflammatory pathologies, in their appearance, development, and evolution, needs to be widely clarified and extended with new future research studies in this interesting field.

Finally, several interesting studies on the role of Breg cells in the immune response to cancer have been reported. For example, it has been reported that in lung cancer, IDO produced by tumor cells and myeloid-derived suppressor cells enhanced Breg differentiation and expansion [32].

In addition, in human solid tumors, a subset of CD1d^+^CD38^+^IgM^+^CD147^+^Bregs was also shown to release the serine protease GrB in addition to IL-10 and IDO and to suppress antitumor immune responses [28]. However, in this context, these suppressive effects were ascribed to GrB, and whether CD1d expression by these Bregs also influenced iNKT cells remains to be clarified. Nevertheless, in different cancers, including renal cell carcinoma, bladder, and prostate cancer, a high B cell presence within the tumor microenvironment is linked to the worst survival prognosis [33,34,35]. However, B cells exhibit contradictory roles within the same type of carcinoma, leading to conflicting findings about their impact on tumor appearance, development, and progression. In this regard, in ductal breast cancer, for instance, elevated CD138^+^PCs were also associated with reduced survival [36]. Conversely, enhanced expression of B cell-related genes, as the Cancer Genome Atlas revealed, correlated with improved survival without metastasis [33]. This divergence underscores the dual nature of B cells in cancer dynamics and collectively demonstrated that intratumoral IL-10^+^ Bregs suppressed CD8^+^ T-cell infiltration and cytotoxic function while promoting the induction of Foxp3^+^Tregs within the tumor [37].

In the end, IL-35^+^ Bregs hindered the infiltration of CD8^+^ T cells, reduced TNF and IFN-γ cytokines production by CD4^+^ T cells, and promoted the accumulation of Foxp3^+^Tregs within the tumor [38,39]. Accordingly, several studies in breast carcinoma have also collectively visualized that TGFβ^+^Breg cells promoted intratumoral Foxp3^+^Treg generation whilst suppressing antitumor T-cell responses [40,41].

As the facts reported and discussed here could be related to modulating tumorigenesis or metastasis, it remains imperative to clarify the exact role of Breg cells in more in-depth studies in the immediate future.

In addition, other processes where B cells, and especially Breg cells, are involved are hematological diseases, immunodeficiencies, viral, bacterial, and parasitic infections, and allergic processes. For example, in immunodeficiencies, B cell dynamics in inborn errors of immunity and immune dysfunction have been noted [42,43,44].

Concerning allergies, there are several studies on atopic dermatitis or food allergies in this context [45,46]. Atopic dermatitis patients displayed a higher percentage of IL-10-producing Breg cells, indicating that these cells promote the differentiation of B cells into Bregs, which produce more anti-inflammatory IL-10, thus alleviating atopic dermatitis symptoms [45]. Bregs also play an immunoregulatory role during eosinophilic esophagitis by controlling esophageal eosinophilia but are functionally impaired due to IFNγ-mediated signaling [46].

With respect to viral, bacterial, and parasitic infections, multiple studies have associated them with B and Breg cells [47,48,49,50,51,52,53]. First, Breg cells modulate immune responses during infection to prevent excessive immune activation [48]. A high percentage of Bregs is positively associated with viral and bacterial load and can contribute to poor vaccine responses [49]. Indeed, Bregs can also facilitate pathogen survival at an early stage of infection and subsequently cause increased severity of disease by inhibiting pro-inflammatory cytokine production, macrophage activation, and inflammatory T cell activation (such as Th1, Th17, and Th22) [49,50]. Also, Bregs afford protection against the hyper-inflammatory response in parasitic infections. In a recent study, chronic Litomosoides sigmoiditis filarial infection was associated with these cells, and in other parasitic infections, such as Leishmania, Baberia, Echinococcus, Helmints, Plasmodium, Schistosoma, Trypanosoma, and Toxoplasma, these cells have also been suggested to play an important role in the infectious response [47,48,49].

Finally, other studies have also suggested that Bregs are important players in cutaneous immunity, and their role in skin immunity, both in healthy states and in pathologies (diabetes, psoriasis, systemic sclerosis, cutaneous lupus erythematosus, cutaneous hypersensitivity, pemphigus, and dermatomyositis), has recently been discussed. Studies have revealed the mechanisms by which these cells maintain tissue homeostasis in the wound microenvironment through the promotion of angiogenesis, suppression of effector cells, and induction of regulatory immune cells [54,55]. Accordingly, IL-10 from Breg cells alleviates atopic dermatitis by suppressing eosinophil activation and tissue infiltration. This elucidates a novel regulatory mechanism of Breg cells, providing a foundation for future Breg-mediated therapeutic strategies for atopic dermatitis, as discussed in a recently published paper [55].

## 4. Regulatory B Cells in Kidney Transplantation

Firstly, allograft rejection can affect any solid organ transplantation, and the role, influence, and determination of Bregs in the post-transplantation period have not been studied.

Secondly, in the field of transplantation, there are very interesting data in animal models of Bregs, where after a combination of CD40L, IL-2, IL-4, and IL-10, it was possible to generate expanded human Bregs (expBregs) with upregulated Tim-1 expression, which managed to suppress the proliferation of CD4^+^ T lymphocytes and the production of TNF and IFN-γ [42]. Blocking Tim-1 in expBregs reduced their ability to suppress T lymphocyte proliferation and cytokine production, independently of IL-10, and the transfer of human expBregs with PBMC to a humanized murine allograft model managed to prolong graft survival and increase Treg infiltration compared to control mice, which was independent of IL-10 [42]. Thus, in mice, Tim-1 expression by B cells is vital for IL-10 production and B cell suppressive capacity, and studies in humans have revealed that Tim-1 acts as a primary suppressor mechanism of Bregs.

However, most studies in clinical practice are performed on kidney allograft transplant recipients, as it is the most frequent form of solid organ transplantation in humans.

In 2021, Alfaro et al. conducted a study to evaluate the evolution of distinct B cell subpopulations in renal transplant recipients by flow cytometry and assess their association with the risk of rejection and graft survival [15].

To do so, they performed two types of analysis: a traditional one, where each subpopulation was examined individually, and a novel cluster analysis, where all B subpopulations were first considered together to establish phenotypic profiles and then compared. Using the classical approach, they observed that two B cell subsets were correlated with renal function after three months post-transplant, with patients with higher values of transitional and class-switched B cells showing better glomerular filtration rates and lower creatinine levels. Nevertheless, they did not find significant differences in the transitional compartment or any other regulatory B cell-enriched subset when comparing patients diagnosed with acute rejection and the control group [15]. These findings are consistent with other published results.

In this context, Guzel et al. did not find statistical differences in any Breg subpopulation, including immature/transitional B cells, plasmablast cells, B10 cells, and BR1 cells in patients with chronic active antibody-mediated rejection [56]. Another recent study showed no relevant variations in B cell subset frequencies (several types of Bregs included), between distinct rejection groups, or in the non-rejection group in a cohort of 1095 patients [57]. Similarly, another study published just this year found that B cell phenotypes in pre-transplant samples of kidney transplant recipients did not reveal any statistical associations between rejection and non-rejection subjects for transitional B cells [58].

On the contrary, when applying their novel cluster analysis, significant changes in the incidence of rejection and graft function were detected by Alfaro et al. between the three main clusters after three months post-transplant [15]. Patients belonging to cluster B2, with a B phenotype enriched in transitional B cells and plasmablasts, exhibited superior glomerular filtration rates and lower creatinine levels compared to clusters B1 (high memory/naive ratio and intermediate levels of transitional cells) and B3 (abundant naïve cells and low transitional cells and plasmablasts). Furthermore, cluster B3 patients had the highest risk of rejection, with all the patients diagnosed with rejection belonging to this group, while cluster B2 patients appeared to have a protective effect. Cluster B1 patients could be considered to have intermediate features, with a lower risk of rejection than cluster B3 but worse transplant outcomes than cluster B2. This group also demonstrated a positive correlation between Breg and Treg values, another lymphocyte subpopulation considered important for graft tolerance in solid organ transplantation [15]. Similar results were shown by a later study using cluster analysis, where cell phenotypes enriched in transitional B cells were significantly reduced in patients with DSAs developing antibody-mediated rejection (ABMR). Interestingly, the study also found differences between groups using traditional analysis, with patients with DSA and ABMR displaying the lowest numbers of transitional B cells, followed by patients with DSA but not ABMR and, finally, DSA-negative patients [59].

Controversial results about the evolution of Bregs after transplantation and to what extent they are implicated in graft tolerance or rejection are abundant in the available literature. For example, Perez-Payá et al. [60] observed that transitional B cells at three months post-transplant were significantly higher in patients with stable graft function than in those with altered graft function. However, they failed to see any statistical differences distinguishing patients with biopsy-proven rejection from those with no signs of rejection [60]. However, another study described transitional B cells as lower in AMR patients than in healthy controls and stable transplanted subjects [61]. In a prospective study by Shabir et al., a protective effect from acute rejection was observed when rejection-free survival curves were estimated for tertiles of transitional B cell frequencies [62]. All patients with less than 3% of transitional B cells exhibited no rejection, while 50% of patients with less than 1% of this subpopulation subset experienced a rejection episode.

A standard limitation for all the studies mentioned above is that their analyses were performed on the peripheral blood compartment, which may not reflect the intra-graft immune microenvironment. To complement this work, in addition to peripheral blood, Luo et al. analyzed the bio-distribution of B cells and IL-10 in kidney grafts of healthy controls, stable transplanted recipients, and ABMR^+^ patients [63]. They observed that while IL-10-producing memory and transitional Bregs decreased in the blood of ABMR^+^ patients, they increased in the graft, with ABMR^+^ patients exhibiting higher IL-10 and CD19 count scores than the other two groups [63]. In contrast to these results, a study using mouse kidney allograft models found an increased expression of Breg markers in accepted kidneys, while minimal expression was seen in the rejection model [64].

Finally, other research groups have also included operational tolerance patients—those who maintain good long-term renal function after complete immunosuppression withdrawal—in their analyses for comparison. For instance, Süsal et al. showed that patients with operational tolerance had significantly higher levels of circulating Tregs and Bregs than all other transplanted groups, independently of the immunosuppression regimen [65].

## 5. Regulatory B Cells in the Transplantation of Other Solid Organs 

Despite most available data on Bregs and transplantation from kidney transplant studies, the evidence suggests that Breg cells may influence the outcome and success of transplantations of other solid organs, such as liver, lung, or heart allografts.

The liver is known to be an immunotolerant organ, yet rejection still happens, although less frequently. Zhou et al. observed that the percentages of memory Bregs were reduced in patients diagnosed with acute rejection compared to those with stable function [66]. Interestingly, these percentages increased after patients received anti-rejection treatment. Transitional Bregs were measured as well, but no significant changes were found. Indirect results from other groups also suggest a role for Bregs in liver transplant tolerance and rejection, as an expansion of transitional B cells and Tregs was seen during the first month in patients converting from Tacrolimus to Sirolimus, a drug reported to induce long-term immunotolerance [67].

In contrast to the liver, rejection rates in lung transplantation recipients are quite high, with nearly 50% of patients developing chronic rejection at 5 years post-transplantation. A study involving 117 lung transplantation recipients supports the participation of transitional Bregs in long-term graft acceptance, as a slight decrease in this cell subset was observed in patients with chronic rejection [68]. Lower frequencies of transitional Bregs were also detected in a later study in the peripheral blood of patients with acute rejection compared to chronic rejection and stable transplanted groups. No significant differences in Bregs were found when comparing bronchoalveolar lavage cells among the three groups, while acute rejection patients showed the lowest levels of CD1d^hi^CD5^+^ and memory Bregs [69].

Regarding heart transplantation, no direct studies linking Bregs to rejection and graft survival have been conducted. Nevertheless, the adoptive transfer of Bregs in heart transplant mouse models was seen to improve graft survival, reduce pro-inflammatory cells, and favor the expansion of Tregs [70]. Furthermore, treatment of heart-transplanted mice with a histone deacetylase inhibitor led to Breg proliferation and prolonged survival rates [71].

Several studies for allogenic islet transplantation have also been conducted on mouse models. Lee et al. established that adoptively transferred Bregs promote tolerance and improve graft survival by inducing Tregs in a TGF-β-dependent way [72]. Another study showed that B10 cells increased in the first week post-transplantation in transplanted mice but returned to normal levels over time, indicating that they may play a crucial part only in the early stage of transplantation tolerance induction. Meanwhile, CD19^+^TIM cells remained elevated for months after transplantation, suggesting a long-term role in tolerance induction and maintenance [73].

Finally, an important limitation of our review is that some articles describe the participation of transitional B cells, not Bregs, in engraftment, although these terms are not identical and could generate confusion. However, the transitional immature B cell compartment is considered vastly enriched in Bregs and is generally used in transplant monitoring.

## 6. Conclusions and Future Directions

Bregs are emerging as an innovative biomarker in transplantation due to their contribution to immune homeostasis by suppressing pro-inflammatory responses and the induction of tolerance. As described above, many studies have found a correlation between the frequency and evolution of different Breg subsets post-transplantation and the incidence of rejection. Although some results seem contradictory and require further clarification, these findings open the door to the idea that Bregs could be used as a prognostic factor in the future to risk-stratify patients and predict rejection episodes before clinical manifestations.

Furthermore, developing therapies aiming to boost Breg expansion or function could help induce tolerance, treat rejection, and minimize immunosuppressive regimens, which often have secondary effects that impact graft survival and an individual’s quality of life, such as organ toxicity and a higher risk of infections. An ongoing clinical trial has demonstrated that the administration of living donor-derived modified immune cells can induce long-lasting B cell-mediated immunotolerance in recipients when given before kidney transplantation. A significant increase in Bregs was seen in these patients, with a 75-fold and 7-fold higher number of transitional Bregs than in 12 long-term survivors on minimal immunosuppression and 4 operationally tolerant patients, respectively [74]. A new era in transplant immunology is coming, but before it becomes a reality, some questions must be resolved. Standardized markers need to be found, molecular pathways behind their origin and function need to be discovered, and the stability, reproducibility, and safety of Breg-based cell therapy need to be investigated more extensively and profoundly in the future.

## Figures and Tables

**Table 1:** Different regulatory B cell subtypes and possible human functions, as published in scientific reports.

Cellular Subtypes	Common Immunophenotype by Flow Cytometry (FC)	Possible Proposed and Reported Functions	References
Transitional	CD19^+^CD24^hi^CD38^hi^	Inhibition of CD4^+^ T cell proliferation. Inhibition of CD4^+^ T cell IFN-γ and TNF-α production. Induction of Treg differentiation. Suppression of monocytes and pDCs IFN-α production.	[11,13,16]
B10 cells	CD24^hi^CD27^+^CD48^+^	Suppression of effector CD4^+^ T cells’ proliferation. Suppression of TNF-α production in monocytes and DCs. Propensity for co-expression with other cytokines such as TGF-β and GrB.	[11,13,16]
Plasmablasts	CD19^+^CD24^hi^CD27^int^CD138 ^hi^Blimp1^+^	Production of IL-10 and TGF-β cytokines. Suppression of DCs and effector CD4^+^ T cells. Suppression of CD4^+^ T cell IFN-γ and TNF-α production.	[11,13,16,17]
Memory	CD19^+^CD24^+^CD27^+^IgM^+^	Suppression of CD4^+^ T cell pro-inflammatory cytokine production. Failure to suppress Th1 responses in particular diseases.	[11,13,16,17,18]
Marginal zone B cells	CD19^+^CD21^hi^CD23^−^	Production of IL-10 cytokine and induction of Treg cells. Suppression of effector CD4^+^ and CD8^+^ T cells.	[11,13,17,18]
BR1 cells	CD19^+^CD25^hi^CD71^hi^CD73^lo^ IL-10^+^	Production of IL-10 cytokine and promotion of IgG4 production. Suppression of inflammatory responses as Th2/Th17. Induction of Foxp3^+^ Treg cells and expansion.	[11,16]
GrB^+^ B cells	CD19^+^CD38^+^CD1d^+^IgM^+^CD147^+^ CD307b^hi^CD258^hi^CD72^hi^CD21^lo^PD-1^hi^	Production of granzyme B. Degradation of TCR ζ chain. Inhibition of CD4^+^ T cell proliferation and Th1 and Th17 responses.	[11,16,18]
CD9^+^ B cells	CD19^+^CD9^+^	Production of IL-10 cytokine Suppression of Th2 and Th17 inflammation	[11,16]
PD-L1^hi^ B cells	CD19^+^PD-L1^hi^	Suppression of circulating Tfh cells	[11,19]
CD5^+^CD1d^+^ cells	CD19^+^CD5^+^CD1d^hi^	Production of IL-10 cytokine Suppression of Th17 response	[11,16]

CD, cluster of differentiation; DC, dendritic cell; FC, flow cytometry technique; Foxp3, Forkhead box P3 (Treg transcription factor); Ig, immunoglobulin; IL, interleukin; TCR, T cell receptor; TGF, transforming growth factor; Th, T helper; Tregs, regulatory T cells.

**Table 2:** Different pathologies in which B cells could be involved and possible functions in humans.

General Processes	Pathologies		References
Autoimmune diseases	Systemic lupus erythematosus Multiple sclerosis Rheumatoid arthritis	Neuromyelitis optica Myasthenia gravis Type 1 diabetes	[19,20,21,22,23,24,25,26,27,28,29,30,31]
Cancers and hematological diseases	Lung cancer Acute lymphocytic leukemia Chronic lymphocytic leukemia Hodgkin lymphoma Non-Hodgkin lymphoma B cell lymphoma Waldenström’s macroglobulinemia Multiple myeloma	Renal carcinoma Prostate cancer Breast cancer Bladder cancer Hepatoma Pancreatic cancer Breast cancer Tumorigenesis and metastasis	[32,33,34,35,36,37,38,39,40,41]
Immunodeficiencies	Inborn errors of immunity Common variable Immunodeficiency Agammaglobulinemia	Hypogammaglobulinemia Hyper-IgE syndrome Selective IgA deficiency	[42,43,44]
Allergy	Atopic dermatitis Food allergy	Altered IgE responses Asthma and rhinitis	[45,46]
Viral and bacterial infections	HBV COVID-19	*Mycobacterium* spp. Helicobacter pylori	[47,48,49,50,51]
Parasitic infections	Filarial infection, Leishmania Baberia, Echinococcus Helminths	Plasmodium, Schistosoma Trypanosoma, Toxoplasma	[47,48,49]
Transplant responses and rejection	Acute antibody-mediated rejection Chronic humoral rejection Desensitization procedures Graft survival and protection Renal and pancreatic islet cell transplants	Heart and lung transplant Liver transplant Intestine transplants Operational tolerance vs. non-tolerance	[52,53,54,55,56,57,58,59,60,61,62,63,64,65,66,67,68,69,70,71]

CF, flow cytometry technique; DC, dendritic cell; TGF, transforming growth factor; IL, interleukin; TCR, T cell receptor; Th, T helper; CD, cluster of differentiation; Ig, immunoglobulin; Tregs, regulatory T cells.

## Data Availability

No new data were created or analyzed in this study. Data sharing is not applicable to this article.

## Abbreviations

The following abbreviations are used in this manuscript:

ABMR	Antibody-mediated rejection
AMR	Antibody-mediated rejection
APRIL	A Proliferation-Inducing Ligand
BAFF	B-cell Activating Factor
BCR	B-cell receptor
BMDM	Bone Marrow-Derived Macrophage
Bregs	Regulatory B cells
BR1	IL-10–producing Regulatory B Cells that promote IgG4
CD	Cluster of Differentiation
CF	Flow Cytometry
CTL	Cytotoxic T Lymphocyte
DC/DCs	Dendritic Cell(s)
DSA	Donor-specific antibodies
expBregs	Expanded Regulatory B Cells
FasL	Fas Ligand
FO	Follicular B Cell
Foxp3	Forkhead box P3 (Treg transcription factor)
GABA	Gamma-Aminobutyric Acid
GITRL	GIT Receptor Ligand (G protein-coupled receptor kinase-interacting protein receptor ligand)
GrB	Granzyme B
HBV	Hepatitis B Virus
HLA	Human Leukocyte Antigens
IDO	Indoleamine 2,3-Dioxygenase
IFN	Interferon
Ig	Immunoglobulin
IgD	Immunoglobulin D
IgE	Immunoglobulin E
IgG	Immunoglobulin G
IgM	Immunoglobulin M
IL	Interleukin
iNKT	Invariant Natural Killer T
ITIM	Immunoreceptor Tyrosine-based Inhibition Motif
LAG3	Lymphocyte Activation Gene 3
MZ	Marginal Zone (B Cell)
MZP	Marginal Zone Precursor (B Cell)
PBMC	Peripheral Blood Mononuclear Cell
PC	Plasma Cell
PD-1	Programmed Cell Death Protein 1
PD-L1	Programmed Death-Ligand 1
pDC	Plasmacytoid Dendritic Cell
SLE	Systemic Lupus Erythematosus
T2-MZP	Transitional-2 Marginal Zone Precursor
TCR	T-cell receptor
Tfh	T Follicular Helper (Cell)
TGF	Transforming Growth Factor
Th	T Helper (Cell)
TIGIT	T Cell Immunoreceptor with Ig and ITIM Domains
Tim-1	T-cell Immunoglobulin and Mucin Domain 1
TLR	Toll-like receptors
TNF	Tumor Necrosis Factor
Tr1	Type 1 Regulatory T Cell
Tregs	Regulatory T Cells
US	United States
